# Harnessing Molecular Phylogeny and Chemometrics for Taxonomic Validation of Korean Aromatic Plants: Integrating Genomics with Practical Applications

**DOI:** 10.3390/plants14152364

**Published:** 2025-08-01

**Authors:** Adnan Amin, Seonjoo Park

**Affiliations:** Department of Life Sciences, Yeungnam University, Gyeongsan 38541, Republic of Korea; adnan.amin@yu.ac.kr

**Keywords:** endemic plants, aromatic, DNA barcoding, molecular phylogenetics, chemotaxonomy

## Abstract

Plant genetics and chemotaxonomic analysis are considered key parameters in understanding evolution, plant diversity and adaptation. Korean Peninsula has a unique biogeographical landscape that supports various aromatic plant species, each with considerable ecological, ethnobotanical, and pharmacological significance. This review aims to provide a comprehensive overview of the chemotaxonomic traits, biological activities, phylogenetic relationships and potential applications of Korean aromatic plants, highlighting their significance in more accurate identification. Chemotaxonomic investigations employing techniques such as gas chromatography mass spectrometry, high-performance liquid chromatography, and nuclear magnetic resonance spectroscopy have enabled the identification of essential oils and specialized metabolites that serve as valuable taxonomic and diagnostic markers. These chemical traits play essential roles in species delimitation and in clarifying interspecific variation. The biological activities of selected taxa are reviewed, with emphasis on antimicrobial, antioxidant, anti-inflammatory, and cytotoxic effects, supported by bioassay-guided fractionation and compound isolation. In parallel, recent advances in phylogenetic reconstruction employing DNA barcoding, internal transcribed spacer regions, and chloroplast genes such as *rbcL* and *matK* are examined for their role in clarifying taxonomic uncertainties and inferring evolutionary lineages. Overall, the search period was from year 2001 to 2025 and total of 268 records were included in the study. By integrating phytochemical profiling, pharmacological evidence, and molecular systematics, this review highlights the multifaceted significance of Korean endemic aromatic plants. The conclusion highlights the importance of multidisciplinary approaches including metabolomics and phylogenomics in advancing our understanding of species diversity, evolutionary adaptation, and potential applications. Future research directions are proposed to support conservation efforts.

## 1. Introduction

Aromatic plants have been known since ancient times for producing essential oils (aromatic components) and have several medicinal applications [1]. These plants are distributed across various geographical regions, with species adapted to temperate and tropical climates [2]. Temperature and soil composition further affect specific volatile compound biosynthesis [3,4]. These variations in environmental factors significantly influence the chemical diversity of essential oils, offering opportunities to optimize cultivation strategies for commercial applications in the pharmaceutical, cosmetic, and food industries [5]. The global essential oils market size was valued at USD 23.74 billion in 2023 and is projected to expand at a compound annual growth rate of 7.6% from 2024 to 2030 [6].

Located in the temperate zone of East Asia, Korea possesses a rich flora characterized by a significant proportion of endemic species that have evolved in response to its complex topography, climatic variation, and geographic isolation by surrounding seas and mountain ranges [7,8]. The Korean Peninsula serves as a biogeographical nexus, integrating elements of northern temperate and subtropical floras while also supporting a unique assemblage of species restricted to its territory [9]. A recently published study shows that the Korean Peninsula harbors 373 endemic taxa, 304 species, 6 subspecies, 49 varieties, and 14 nothospecies distributed across 179 genera and 64 families. These endemics represent 9.5% of the total native flora of the region. The most represented families include *Asteraceae*, *Ranunculaceae*, *Liliaceae* s.l., and *Rosaceae* (41, 29, 24, and 22 taxa, respectively) [10]. Among these, Korean endemic plants encompass various taxa—including herbaceous species, shrubs, and trees—highlighting the various life forms adapted to ecological niches across the Korean Peninsula [11]. This endemism is particularly pronounced in mountainous regions, where microclimatic and edaphic variations promote speciation and niche specialization [12]. These endemic species hold scientific and cultural value, often associated with traditional knowledge and natural heritage preserved over centuries.

Korean aromatic plants constitute a unique and invaluable component of the rich botanical diversity in the Korean Peninsula [13]. Restricted in distribution exclusively to Korea, these species have evolved under specific ecological and geographical conditions, leading to distinctive chemical profiles and genetic traits that distinguish them from closely related taxa in neighboring regions [14]. The aromatic nature of these species is primarily attributed to the presence of volatile secondary metabolites such as essential oils composed of phenolics, terpenes, aldehydes, and ketones, among others which have attracted significant scientific attention owing to their ecological functions and potential applications in medicine, perfumery, and agriculture [15]. Beyond their ecological and evolutionary significance, aromatic plants serve as reservoirs of bioactive compounds with diverse pharmacological properties [16,17]. Traditional Korean medicine and ethnobotanical practices have long employed several aromatic plants, and contemporary phytochemical research has increasingly validated their therapeutic potential [18,19]. Essential oils extracted from these species exhibit antimicrobial, antioxidant, and anti-inflammatory activities, contributing to their promise as candidates for drug development and natural product-based therapies [20,21].

The conservation status of Korean aromatic plants is increasingly threatened by rising anthropogenic pressures, including habitat fragmentation, overharvesting, and environmental changes induced by global warming [22]. Many of these species are restricted to specialized habitats such as mountainous regions or island ecosystems—making them susceptible to extinction threats [13,23]. Conservation efforts including in situ protection and ex situ cultivation in botanical gardens and seed banks are essential for preserving their genetic diversity and ensuring sustainable utilization [12]. Moreover, integrating chemotaxonomic and phylogenetic information can further support conservation prioritization by identifying evolutionarily distinct and chemically unique taxa [24,25]. Balancing utilization with conservation will require implementing sustainable harvesting practices aligned with traditional knowledge and modern cultivation techniques.

Several national-level floristic resources provide foundational data on Korean plant biodiversity. Notable among these are the Flora of Korea (updated volumes by Korea National Arboretum), the National List of Endemic Species of Korea (https://www.nibr.go.kr/aiibook/catImage/64/endemic%20species%20of%20korea.pdf, accessed on 25 May 2025), and the Illustrated Book of Korean Medicinal Plants published by the Korea Institute of Oriental Medicine [26], which collectively document species distribution, endemism, and traditional uses. Previous reviews on Korean aromatic plants have primarily focused on either ethnopharmacological usage or essential oil chemistry in select genera (e.g., *Artemisia*, *Schisandra*, *Perilla*, etc.). However, these studies tend to lack integrated phylogenetic perspectives or comprehensive chemotaxonomic comparisons. In contrast, the present review distinguishes itself by synthesizing a wide array of data including floristic records, phytochemical profiles, pharmacological activity and molecular phylogenetics to offer a more holistic understanding of Korea’s aromatic flora. By situating the discussion within an evolutionary and conservation framework, this work provides a broader context than earlier studies and identifies research gaps, such as underexplored taxa with high chemical or evolutionary distinctiveness. By critically evaluating the chemical profiles and molecular data, this study hopes to clarify the taxonomic and evolutionary frameworks underpinning these species. Additionally, this review seeks to highlight the pharmacological potential of Korean aromatic plants by integrating experimental findings with ethnobotanical knowledge, while addressing conservation challenges and strategies for sustainable management. This review also emphasizes an integrative perspective that bridges botanical taxonomy, phytochemistry, molecular phylogenetics, and bioactivity, thereby guiding future research and fostering interdisciplinary collaboration to unlock the full potential of aromatic flora in Korea.

## 2. Literature Search and Methodology

**Figure 1:**
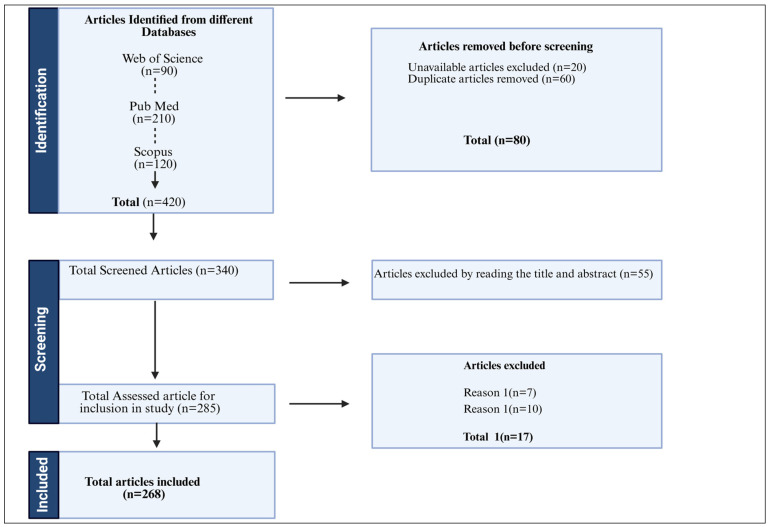
PRISMA based study selection.

The review is based on a systematic and comprehensive analysis of peer-reviewed journal articles, review papers, and authoritative sources relevant to Korean aromatic plants. The detailed literature review was performed by involving the PRISMA (Preferred Reporting Items for Systematic Reviews and Meta-analyses) guidelines, which were conceived to promote a transparent report of results [27]. The PRISMA methodology is divided into four stages: identification, screening, eligibility, and inclusion for analysis [28]. Relevant literature was identified through searches in major academic databases including Web of Science, Scopus, and Pubmed. The search utilized targeted keywords and phrases that was performed to refine searches and have detailed overview (Table 1). The timeframe for included publications primarily ranged from 2001 through 2025 to incorporate foundational studies as well as the latest developments. Inclusion criteria mandated that studies focus on Korean aromatic plants. Exclusions were made for grey literature, non-ecofriendly conference abstracts, and unpublished data to maintain academic rigor. Moreover, those with duplication, those not having database full access, and articles not written in English and falling in Reason-1 (they were not related to Korea in particular) and Reason-2 (having limited information relation to our investigation) were excluded (Figure 1). Preliminarily, a dataset of approximately 420 records was retrieved and curtained by titles and abstracts for relevance and quality. Following this, 340 articles were reviewed in full text, with 268 meeting all criteria for inclusion in the final citation. To appraise study quality, we employed the [ROBIS/SYRCLE] tool to assess the risk of bias and ensured the inclusion of studies with an acceptable risk profile. This approach ensured a wide yet focused coverage of the topic. Figures included in this review were generated using Bio render and Napkin.ai for conceptual illustrations. All contents were carefully vetted to ensure accurate representation of the discussed content.

## 3. Diversity and Distribution of Korean Aromatic Plants

Korean aromatic plants including endemic and non-endemic species, represent a vital component of botanical diversity in the Korean Peninsula, exhibiting unique evolutionary adaptations and occupying ecological niches shaped by the complex geography and climatic history [10,29,30]. These species, restricted exclusively to the Korean Peninsula, thrive across diverse habitats, ranging from high-altitude mountain ranges and temperate forests to coastal zones and volcanic islands [31]. Combining historical isolation, climatic fluctuations during the Quaternary period, and the varied topography across the peninsula has facilitated the speciation and persistence of these aromatic taxa exhibiting distinct genetic and chemical profiles [32]. Recent advances in biogeographical analyses including GIS-based distribution mapping and ecological niche modeling have enhanced our understanding of their spatial patterns and habitat preferences [33].

### 3.1. Geographic Distribution and Habitat Specificity

The geographic distribution of Korean aromatic plants is uneven and strongly influenced by environmental gradients and historical refugia. The Baekdudaegan mountain range, which extends longitudinally across the Korean Peninsula, serves as a major biodiversity corridor and a hotspot for several endemic species, including many aromatic plants [34]. These mountains offer a mosaic of microhabitats characterized by diverse altitudinal zones, temperature regimes, and soil types, enabling niche specialization and adaptive divergence [35]. Several aromatic plant species are restricted to alpine or subalpine zones, where exposure to harsh climatic conditions has driven the evolution of unique physiological and morphological traits associated with aromatic compound production [36]. Coastal areas and islands such as Jeju, Ulleungdo, and Dokdo, among others, serve as additional centers of aromatic plant diversity, supporting species adapted to saline conditions, strong winds, and volcanic soils [37]. These island habitats are also often characterized by high endemism rates owing to geographic isolation, which restricts gene flow and promotes allopatric speciation

Ecological studies reveal that Korean aromatic plants often inhabit specialized environments such as rocky slopes, forest understories, and riparian zones characterized by specific microclimatic conditions, including temperature stability, humidity, and light availability [23,38]. Soil characteristics including pH, nutrient content, and texture significantly influence species distribution, as demonstrated by field surveys correlating soil chemistry with plant occurrence patterns [39].

### 3.2. Aromatic Plants Hotspots

**Figure 2:**
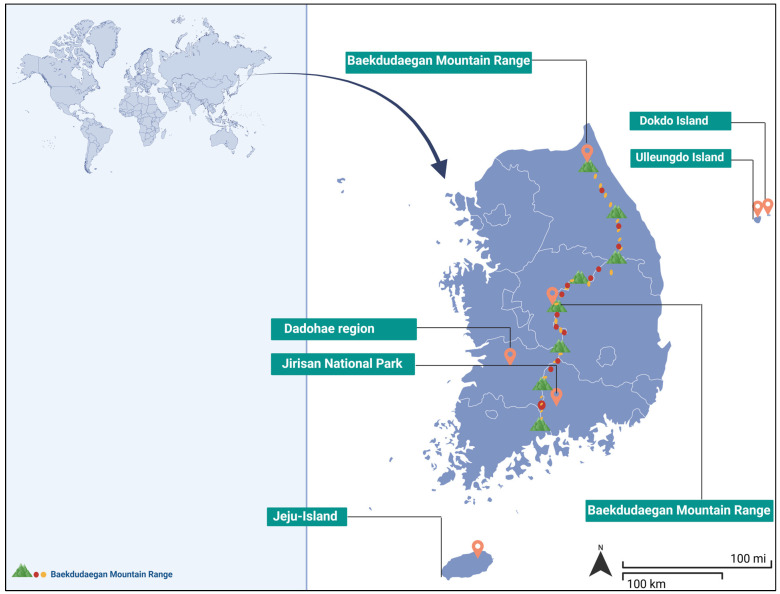
Korean map showing hotspots of endemic aromatic plants across key islands and mountain ranges.

The rich topographical and climatic variation across Korea has led to the emergence of several well-defined hotspots that support endemic aromatic plant species, each characterized by distinct ecological conditions and phytochemical profiles [40]. These hotspots are distributed throughout Korea (Figure 2), with one of the most prominent being Jeju Island, particularly the Hallasan Biosphere Reserve. This region encompasses a broad altitudinal gradient, ranging from sea level to the alpine summit of Mount Hallasan, creating diverse habitats that support subtropical to alpine species [41]. Volcanic soils and humid conditions in Jeju support the growth of aromatic endemic species—such as *Viola acuminata*, *Hemerocallis hakuunensis*, and *Paeonia obovata* var. *koreana*, which are rich in essential oils and phenolic compounds [42]. These species hold cultural significance and exhibit strong antioxidant and antimicrobial activities [43]. However, increasing urban development and ecotourism-related pressures have raised conservation concerns regarding habitat fragmentation and population decline [44].

Another significant hotspot is Ulleungdo Island, a volcanic island in the East Sea, recognized for its high level of endemism and ecological isolation [45]. The island supports approximately 30 endemic plant species, many of which exhibit aromatic properties [45]. Notable examples include *Allium ulleungense*, a wild onion rich in high sulfur-containing volatile content [46], *Senecio ulleungensis*, and local *Artemisia* variants rich in sesquiterpene content [47]. These plants have adapted to the steep terrain, maritime climate, and high humidity of Ulleungdo Island, often evolving specialized defense-related volatile compounds [48]. Limited gene flow and susceptibility to invasive species make Ulleungdo Island a high-priority site for biodiversity conservation and research on aromatic plants [49].

The Baekdudaegan Mountain Range, which forms the ecological backbone of the Korean Peninsula, also plays a central role in shaping the distribution of endemic aromatic flora [40]. Extending from north to south, this extensive range encompasses diverse microclimates, altitudes, and forest types, creating favorable conditions for species diversification [36,50]. Endemic aromatic plants—including several *Corydalis* and *Pinus* spp.—are present in this region, exhibiting notable variability in their phytochemical profiles depending on elevation and habitat [10,51]. These species produce volatile oils and are commonly used in traditional medicine [52]. As climate change continues to alter altitudinal vegetation zones, shifts in species distribution have become increasingly evident, emphasizing the need for ecological monitoring and climate-adaptive conservation strategies regarding the Baekdudaegan Mountain Range [53].

In southern Korea, Jirisan National Park serves as a botanical refuge and contains one of the highest levels of floral diversity in the country [54]. As the southern terminus of the Baekdudaegan range, Jirisan supports a mosaic of deciduous and coniferous forests that provide suitable habitats for several aromatic endemic species [55]. The Jirisan mountain range serves as a rich source of several endemic plants, including *Fraxinus chiisanensis*, *Filipendula formosa* (aromatic), *Rhododendron tschonoskii*, *Pseudostellaria okamotoi*, and *Arundinaria munsuensis* [54,56]. These are recognized for their medicinal properties, owing to their extensive secondary metabolite profiles that support their traditional use in Korean medicine as anti-inflammatory, anticancer, and detoxifying agents [57]. Conservation infrastructure within the park, along with its cultural significance, has supported relatively stable population dynamics. However, threats from illegal harvesting and tourism persist [58].

Seoraksan and Odaesan National Parks, located in the northeastern alpine zone of Korea, represent another critical region for cold-adapted aromatic endemic species [59]. These mountainous regions support species such as *Pseudostellaria seoraksanensis*, *Pseudolysimachion kiusianum*, and *Saussurea diamantiaca Nakai* [60], which contain compounds that fulfill ecological functions, including herbivore deterrence and microbial resistance [61]. These regions hold significant value for scientific research, particularly in understanding how plants modify chemical defense strategies in response to extreme climatic conditions. Given their popularity as tourist destinations, implementing careful zoning and ecosystem protection measures is necessary to prevent habitat degradation [62].

Lastly, Dokdo Island—despite its small size and relatively low overall biodiversity is recognized as a micro-endemic site of ecological significance [49,63]. While its floral diversity is limited compared to that of larger ecosystems such as Jeju or Ulleung Island, Dokdo Island can still be considered a minor hotspot for endemic plant species. From a scientific perspective, Dokdo (also known as the Liancourt Rocks) holds ecological significance owing to extreme isolation, harsh volcanic terrain, and maritime climate, all of which have contributed to the presence of uniquely and highly adapted plant species [37]. Located in the East Sea, its steep volcanic cliffs, limited soil cover, and harsh saline conditions have led to the development of highly specialized aromatic flora such as *Phedimus takesimensis* [64], along with various mosses and lichens [37,65]. These species exhibit strong resilience to environmental stress, often producing unique volatile compounds that aid in regulating water loss or deterring marine herbivores. Despite its limited diversity, the ecological isolation of Dokdo and its sensitivity to climate change make it an important site for long-term biodiversity and plant monitoring [66]. Including Dokdo in the national conservation framework emphasizes the importance of protecting not only floristically rich regions but also microhabitats where evolutionary processes actively shape rare and resilient plant life.

### 3.3. Taxonomic Diversity of Aromatic Endemic Species

In total, >300 plant species are classified as endemic to Korea, many of which produce essential oils or other aromatic compounds [15,67]. The taxonomic diversity of Korean endemic aromatic plants spans multiple families, with *Lamiaceae*, *Asteraceae*, *Rosaceae*, Ranunculaceae, and Papaveraceae being particularly prominent owing to their abundant production of essential oils and diverse secondary metabolites, including terpenoids, phenolic compounds, aldehyde alkaloids, flavonoids, and saponins [31,32,68]. These families collectively contribute to the rich chemical and ecological diversity observed uniquely within endemic flora across Korea, reflecting long-term evolutionary adaptation to distinct climatic zones, altitudinal gradients, and geographically isolated habitats across the peninsula [30,32]. Among them, *Lamiaceae* emerges as one of the most prominent families of Korean endemic aromatic species, characterized by herbs and shrubs that produce complex essential oils rich in monoterpenes and sesquiterpenes [69,70]. Endemic species in Korea within this family such as *Thymus quinquecostatus Celak*, *Thymus magnus*, and *Agastache rugosa* are well-known for their fragrant foliage and flowers [71,72]. These species have been documented to exhibit antioxidant, antimicrobial, and anti-inflammatory bioactivities [72,73] that support their traditional uses and highlight their ecological significance within Korean ecosystems.

Within Korean endemic aromatic flora, Asteraceae and Rosaceae are represented by species that produce diverse secondary metabolites, including essential oils, sesquiterpene lactones, alkaloids, and glycosides [74]. The genus *Artemisia* is particularly known for its aromatic members [75]. Among Korean endemics, *Artemisia japonica* ssp. *littoricola* is restricted to Dokdo Island and has been studied for its medicinal and aromatic properties, including antioxidant and insecticidal activities [76].

The *Rosaceae* family is primarily known for its morphological and ecological diversity. Among Korean endemic aromatic plants, species such as *Rubus coreanus* and *Cotoneaster wilsonii* have been reported. Both species are reported to contain terpenoids, phenolics, as well as aldehydic and alcoholic compounds that contribute to various biological activities [77,78]. These species exemplify the morphological and chemical diversity shaped by the complex mountain ecosystems and microhabitats found across Korea, highlighting patterns of adaptive radiation and ecological specialization within the peninsula. Similarly, Species from the Pinaceae family including *Pinus densiflora* (Japanese red pine) and *Pinus parviflora* (Japanese white pine) are commonly found in the mountainous regions of Korea, including the Baekdudaegan Mountain Range and Jirisan National Park. These species are renowned for their aromatic properties [79], with essential oils rich in terpenes such as α-pinene. Traditionally, they have been used in Korean medicine for antiseptic, anti-inflammatory, and respiratory health benefits, while also serving as important resources in the fragrance industry [80]. The Lauraceae family recognized for its aromatic evergreen species—also includes trees and shrubs that produce essential oils rich in terpenes, phenols, and other bioactive compounds, making them valuable in medicinal and commercial applications [81]. Species from the Lauraceae family, such as *Neolitsea sericea* and *Machilus japonica*, occurring in the temperate regions of Korea, including Jeju Island and Jirisan National Park [82], are recognized for their aromatic properties. Their essential oils contain compounds such as linalool and eugenol, and these species have been traditionally used in Korean medicine for antimicrobial, anti-inflammatory, and therapeutic effects while also contributing to the fragrance industry [83]. Table 2 shows a detailed overview of Korean endemic aromatic plants.

The convergent evolution of aromaticity across these taxonomic groups illustrates how different lineages endemic to Korea have independently developed similar chemical defenses and pollinator attractants, enriching the botanical heritage unique to the Korean Peninsula [84,85]. Ongoing taxonomic revisions that employ integrative approaches—combining morphology, chemotaxonomy, and molecular phylogenetics are essential to fully resolve the diversity and evolutionary relationships among these endemic aromatic species [86]. Comprehensive knowledge of these species will support effective conservation and sustainable use strategies, which are essential for safeguarding these irreplaceable plant resources amidst environmental change and increasing anthropogenic effects in Korea.

**Table 2:** Key Korean endemic aromatic plants with their major geographical distribution.

Region/Location	Species Name	Family Name	Part Used	Nature of Plants	Citation
Jeju Island/Hallasan Biosphere Reserve	*Pinus thunbergii* Parl.	Pinaceae	Wood and resin	Non-endemic	[87]
	*Abies koreana* E.H. Wilson	Pinaceae	Wood and needles	Endemic	[88]
	*Machilus japonica* Siebold and Zucc.	Lauraceae	Bark and leaves	Non-endemic	[89]
	*Cinnamomum camphora* (L.) J. Presl	Lauraceae	Leaves and bark	Non-endemic	[90]
	*Cinnamomum loureirii* Nees	Lauraceae	Leaves and bark	Non-endemic	[90]
	*Neolitsea sericea* (Blume) Koidz.	Lauraceae	Leaves and bark	Non-endemic	[83]
	*Zanthoxylum ailanthoides* Siebold and Zucc.	Rutaceae	Fruit and bark	Non-endemic	[91]
	*Citrus reticulata* Blanco.	Rutaceae	Fruit and peel	Non-endemic	[92]
	*Citrus unshiu* (Yu. Tanaka ex Swingle) Marcow.	Rutaceae	Fruit and peel	Non-endemic	[93]
	*Zanthoxylum coreanum* Nakai	Rutaceae	Fruit and bark	Non-endemic	[94]
	*Cryptomeria japonica* (Thunb. ex L.f.) D. Don	Cupressaceae	Wood and bark	Non-endemic	[94]
	*Agastache rugosa* (Fisch. and CA Mey.) Kuntze	Lamiaceae	Leaves and flowers	Non-endemic	[91]
	*Magnolia kobus* DC.	Magnoliaceae	Bark and flowers	Non-endemic	[94]
	*Vitex rotundifolia* L.f.	Verbenaceae	Leaves and flowers	Non-endemic	[94]
	*Chamaecyparis pisifera* (Siebold and Zucc.) Endl.	Cupressaceae	Wood and bark	Non-endemic	[94]
	*Artemisia hallaisanensis*	Asteraceae	Leaves and flowers	Endemic	[95]
	*Artemisia japonica* ssp. littoricola	Asteraceae	Leaves and flowers	Non-endemic	[95]
Baekdudaegan Mountain Range	*Pinus rigida* Mill	Pinaceae	Wood and resin	Non-endemic	[96]
	*Pinus densiflora* for. multicaulis	Pinaceae	Wood and resin	Non-endemic	[97]
	*Pinus parviflora* Siebold and Zucc.	Pinaceae	Wood and resin	Non-endemic	[80]
	*Agastache rugosa* (Fisch. and CA Mey.) Kuntze	Lamiaceae	Leaves and flowers	Non-endemic	[98]
	*Magnolia kobus* DC.	Magnoliaceae	Bark and flowers	Non-endemic	[99]
Jirisan National Park	*Pinus densiflora* for. multicaulis	Pinaceae	Wood and resin	Non-endemic	[80]
	*Abies nephrolepis* (Trautv. ex Maxim.) Maxim.	Pinaceae	Wood and needles	Non-endemic	[100]
	*Pinus parviflora* Siebold and Zucc.	Pinaceae	Wood and resin	Non-endemic	[80]
	*Machilus japonica* Siebold and Zucc.	Lauraceae	Bark and leaves	Non-endemic	[83]
	*Pinus koraiensis* Siebold and Zucc.	Pinaceae	Wood and resin	Non-endemic	[101]
	*Thuja koraiensis* Nakai	Cupressaceae	Wood and bark	Non-endemic	[102]
	*Chamaecyparis pisifera* (Siebold and Zucc.) Endl.	Cupressaceae	Wood and bark	Non-endemic	[102]
	*Juniperus chinensis* L.	Cupressaceae	Wood and leaves	Non-endemic	[91]
	*Tsuga sieboldii* Carriere	Pinaceae	Wood and needles	Non-endemic	[103]
	*Abies holophylla* Maxim.	Pinaceae	Wood and needles	Non-endemic	[91]
	*Artemisia montana* (Nakai) Pamp.	Asteraceae	Leaves and flowers	Non-endemic	[56]
	*Zanthoxylum schinifolium* Siebold and Zucc.	Rutaceae	Fruit and bark	Non-endemic	[104]
	*Artemisia absinthium* L.	Asteraceae	Leaves and flowers	Non-endemic	[56]
	*Rubus coreanus*	Rosaceae	Berries and leaves	Endemic	[104]
	*Juniperus chinensis* L.	Cupressaceae	Wood and leaves	Non-endemic	[54]
	*Artemisia annua* L.	Asteraceae	Leaves and flowers	Non-endemic	[56]
Dokdo Island	*Artemisia japonica* ssp. littoricola	Asteraceae	Leaves and flowers	Non-endemic	[105]
Throughout Korea	*Artemisia annua* L.	Asteraceae	Leaves and flowers	Non-endemic	[54,106]
	*Artemisia capillaris* (Thunb.) Besser	Asteraceae	Leaves and flowers	Non-endemic	[107]
	*Artemisia iwayomogi* Kitamura	Asteraceae	Leaves and flowers	Non-endemic	[107]
	*Artemisia absinthium* L.	Asteraceae	Leaves and flowers	Non-endemic	[107]
	*Thymus quinquecostatus* Celak			Endemic	[108]
Dadohae region/Ulleungdo island	*Artemisia absinthium* L.	Asteraceae	Leaves and flowers	Non-endemic	[47]
	*Pinus densiflora* for. multicaulis	Pinaceae	Wood and resin	Non-endemic	[97]
	*Agastache rugosa* (Fisch. and CA Mey.) Kuntze	Lamiaceae	Leaves and flowers	Non-endemic	[91]
	*Pinus parviflora* Siebold and Zucc.	Pinaceae	Wood and resin	Non-endemic	[80]

## 4. Chemotaxonomy of Aromatic Plants

Chemotaxonomy has emerged as a critical tool in classifying and systematically studying medicinal plants for decades, thereby providing complementary evidence to traditional morphological and molecular taxonomy [25,109]. This approach leverages the diversity and specificity of secondary metabolites particularly essential oils, terpenoids, phenolics, and alkaloids to delineate taxonomic boundaries, trace evolutionary relationships, and reveal cryptic diversity within endemic taxa [110] (Table S1). The chemotaxonomic approach has been instrumental in resolving taxonomic uncertainties where morphological traits are insufficient owing to phenotypic plasticity or convergent evolution [111]. Advances in chromatographic and spectroscopic techniques including gas chromatography-mass spectrometry, high-performance liquid chromatography, liquid chromatography coupled with quadrupole time-of-flight mass spectrometry, matrix-assisted laser desorption/ionization time-of-flight mass spectrometry, and nuclear magnetic resonance spectroscopy have enabled detailed characterization of complex chemical profiles in these species [112,113]. This section presents recent findings on chemical profiles, highlights their taxonomic significance, and explores the integration of metabolomics with classical taxonomy in the study of Korean aromatic flora.

### 4.1. Phytochemical Markers and Chemotaxonomic Classification

Phytochemical markers are specific chemical compounds or classes of compounds found in plants that serve as distinctive indicators for plant identification, classification, and differentiation [114,115]. These markers typically include alkaloids, flavonoids, saponins, essential oils (terpenoids), tannins, and plant steroids, among others [25,116]. This approach is important, as many compound classes exhibit species-specific profiles based on their relative abundance. Several examples illustrate this approach; for instance, the presence of specific sesquiterpene lactones in *Elsholtzia* species (Lamiaceae) serves as a diagnostic feature that distinguishes them from closely related taxa in East Asia [117]. Similarly, the flavonoid composition in the genus *Zanthoxylum* (Rutaceae) provides taxonomically informative patterns that aid in genus- and species-level discrimination [118].

Alkaloid patterns in plant species also contribute significantly to chemotaxonomy [119]. Isoquinoline alkaloids, which are prevalent in families such as Papaveraceae and Rutaceae, exhibit distinct distribution patterns that correspond to taxonomic boundaries [120,121]. For example, the alkaloid profiles of endemic *Corydalis* species serve as diagnostic characters, highlighting evolutionary history and adaptation to local environments [122,123].

Similarly, flavonoids a large group of polyphenolic compounds also serve as important chemotaxonomic markers owing to their distinct chemical structures and variability across plant species. These compounds include flavonols, flavones, anthocyanins, and isoflavones, which are frequently used to differentiate plant species, genera, and families. They are so diverse that in the Rutaceae family alone, >800 compounds comprising 4700 flavonoid occurrences have been documented, highlighting their value as reliable chemotaxonomic markers worldwide [124].

**Figure 3:**
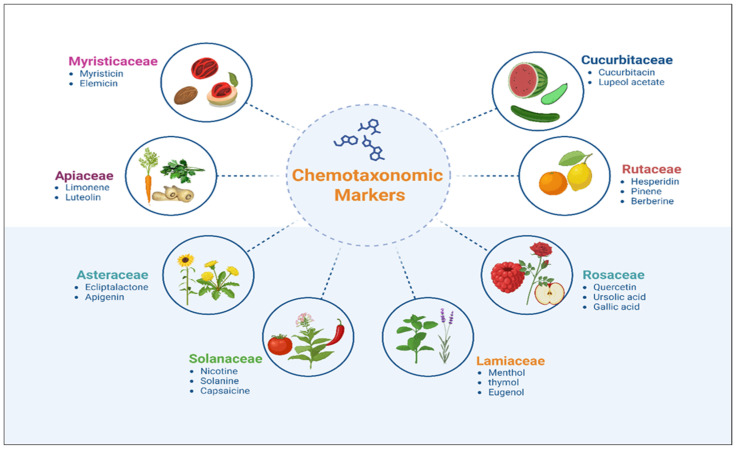
Representative classes of chemotaxonomic marker compounds associated with respective plant families.

Polysaccharides derived from aromatic plants have attracted increasing scientific interest due to their structural diversity and bioactive potential [125]. While research on aromatic plants has historically centered on volatile oil constituents, recent investigations have shifted focus to their non-volatile macromolecules, particularly polysaccharides, which exhibit significant pharmacological and functional properties. These naturally occurring biopolymers, primarily found in plant cell walls and storage tissues, contribute to the therapeutic efficacy of numerous medicinal species [126]. The Zingiberaceae family, comprising over 50 genera and 1300 species, includes several widely utilized medicinal plants such as *Zingiber officinale* (ginger), *Curcuma longa* (turmeric), *Alpinia galanga*, *Kaempferia galanga*, and various *Amomum* species [127]. These taxa are primarily characterized by their aromatic rhizomes, which serve as repositories for bioactive polysaccharides in addition to essential oils (Figure 3).

### 4.2. Essential Oils as Chemotaxonomic Markers

Essential oils aromatic compounds extracted from various parts of plants have long been utilized in traditional medicine, perfumery, and culinary practices [128,129]. In addition to contributing to the characteristic fragrance of these plants, essential oils exhibit various biological activities such as antimicrobial, antioxidant, anti-inflammatory, and anticancer properties [130]. Essential oils have long been recognized as valuable chemotaxonomic markers in plant classification [131]. They are primarily composed of terpenoids, phenylpropanoids, and occasionally other secondary metabolites, all of which exhibit significant variability among plant species [132]. Owing to their chemical complexity and species-specific composition, essential oils offer an effective means of distinguishing between closely related plant taxa [133].

One of the key advantages of using essential oils as chemotaxonomic markers lies in their capability to capture the unique metabolic signature of a plant. Several studies have employed essential oil components as chemotaxonomic markers. For instance, terpenoids, the most abundant group of compounds in essential oils, are particularly valuable in this context [134]. These compounds are synthesized from isoprene units and can be classified into monoterpenes, sesquiterpenes, and diterpenes based on their molecular structure [135]. Similarly, monoterpenes, including limonene and pinene, and sesquiterpenes such as caryophyllene and β-caryophyllene, serve as distinctive markers in many aromatic plants [136]. For example, the genus *Mentha* (mint) is characterized by a high content of menthol and menthone, while *Thymus* (thyme) species are distinguished by the presence of thymol and carvacrol [137]. These chemical distinctions enable precise identification and differentiation of species that may appear morphologically similar [138].

*Artemisia*, a large genus within the Asteraceae family encompasses various aromatic plants, including *Artemisia annua*, *Artemisia japonica*, *Artemisia keiskeana*, and *Artemisia takeshimensis* [139]. These species produce characteristic essential oils, with major components such as 1, 8-cineole, β-pinene, camphene, caryophyllene, thujone, artemisia ketone, camphor, and germacrene D [140]. These chemical profiles are distinct from those of related taxa in neighboring regions [141]. The richness in sesquiterpenes and phenolic compounds serves as valuable chemotaxonomic markers, aiding in species differentiation and reflecting adaptations to specific montane and island habitats [142].

**Figure 4:**
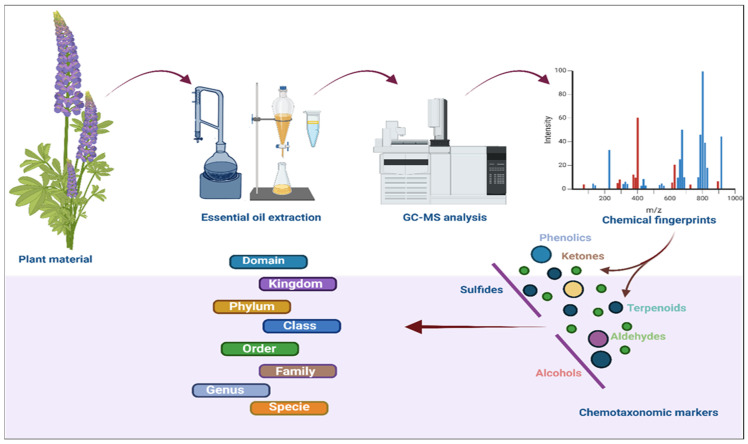
Overview of essential oil-containing plants used as chemotaxonomic markers from extraction to phytochemical analysis and applications.

Another significant example comes from the family Rutaceae, where the essential oils in the *Citrus* genus are particularly rich in monoterpenoids such as limonene, pinene, and myrcene [143]. The composition of these monoterpenes can vary across different *Citrus* species and serves as effective chemotaxonomic markers [144]. Moreover, essential oils can reveal chemotypic variation within a single species, further enhancing their utility as chemotaxonomic markers [145]. Chemotypes are defined as distinct chemical variants within a species that exhibit unique metabolic profiles [146]. The ability to distinguish between chemotypes within a species provides further depth to chemotaxonomic studies, enabling more refined identification at the subspecies or variety level (Figure 4).

### 4.3. Essential Oil Composition

Chemotaxonomy offers valuable insights, particularly in aromatic plant species where essential oils play a central role [112]. These oils are mixtures of volatile secondary metabolites, primarily composed of terpenoids, phenylpropanoids, phenolics, aldehydes, esters, ketones, alkaloids, and sulfur-containing compounds [147].

Terpenoids, also known as isoprenoids, represent a vast and diverse class of plant-derived natural products [130]. These compounds are synthesized from five-carbon isoprene units and can be classified as mono (C-10; e.g., limonene, myrcene, and pinene), sesqui (C-15; e.g., *β*-caryophyllene and farnesene), di (C-20; e.g., taxol and abietic acid), tri (C-30; e.g., lupeol and betulinic acid) and tetra (C-40; e.g., lutein and β-carotene) terpenes [148]. These compounds are biological active and exhibit various chemical structures and biological activities [130]. Terpenoids are essential in plant defense, communication, and overall metabolic processes [149]. They also serve as significant chemotaxonomic markers, helping in the identification and classification of plant species [150]. Figure 4 shows a detailed structural overview.

Phenylpropanoids are a significant class of bioactive compounds commonly found in essential oils. Derived from the phenylpropanoid pathway and synthesized from the amino acid phenylalanine, these compounds play key roles in plant defense and possess numerous medicinal and aromatic properties. Typical examples include cinnamaldehyde and eugenol [151]. In addition, these compounds exhibit various biological activities, including antimicrobial, anticancer, anti-inflammatory, analgesic, and antioxidant properties [152,153].

Phenolic compounds represent a diverse group of plant secondary metabolites that play essential roles in plant defense and contribute to the aroma and biological properties of essential oils [154]. These compounds include phenolic acids, flavonoids, and lignans, all of which contain hydroxyl groups attached to aromatic rings, a feature that confers antioxidant, antimicrobial, and anti-inflammatory properties [155]. Typical examples of phenolic compounds include thymol, resveratrol, and carvacrol, among others. The phenolic content in essential oils can aid in differentiating between varieties within the same species. Moreover, the diversity of phenolic compounds across plant families, e.g., Lamiaceae, Myrtaceae, and Lauraceae enhances their value in plant taxonomy and species identification [156].

Aldehydes are a class of organic compounds characterized by the presence of a carbonyl group (C=O) bonded to a hydrogen atom (H) and a variable side chain or hydrogen [147,157]. Aldehydes are commonly present in essential oils and contribute significantly to their aroma and therapeutic properties. Some key aldehydes present in essential oils include cinnamaldehyde, citral, decanal, dodecanal, and benzaldehyde, among others [151]. These compounds exhibit antimicrobial, antioxidant, anti-inflammatory, and analgesic properties. Some aldehydes, particularly benzaldehyde, also exhibit mild sedative properties [158]. In chemotaxonomy, aldehydes play a significant role in classifying plants based on their chemical composition. The presence or absence of specific aldehydes in essential oils aids in distinguishing between different plant families and genera. For instance, cinnamaldehyde serves as a distinctive marker for species within the *Lauraceae* family, such as *Cinnamomum verum* (cinnamon) [136].

Recent technological advancements in metabolomics, including untargeted profiling and multivariate statistical analyses, have significantly enhanced chemotaxonomic investigations by enabling comprehensive characterization of the metabolite landscape. Studies employing principal component analysis, hierarchical clustering, and partial least squares discriminant analysis (PLS-DA) on chemical datasets have demonstrated clear discrimination among various plant species and even among populations within the same species [159] (Figure S1).

## 5. Pharmacological Effects of Korean Aromatic Plants

Below is a detailed overview of selected Korean aromatic plants, highlighting their major chemical constituents, common occurrence, industrial applications, traditional uses, and pharmacological effects. Table 3 presents a detailed overview of Korean aromatic plants with pharmacological properties.

### 5.1. Thymus quinquecostatus Celak (Lamiaceae; Bak-Ri-Hyang)

*Thymus quinquecostatus* is a small perennial herb native to Korea, commonly found in rocky, well-drained soils of mountainous regions [160]. The plant is characterized by narrow, aromatic leaves with a strong, pleasant fragrance and produces small pink or purplish flowers that bloom in dense clusters [161]. *Thymus quinquecostatus* is known for its adaptability to various environmental conditions, particularly thriving in cold, high-altitude climates. Its essential oil is rich in bioactive compounds such as thymol, carvacrol, cymene, and terpinen-4-ol, which contribute to its distinctive spicy, herbal aroma. This essential oil is highly valued for its antioxidant, antibacterial, antifungal, and anti-inflammatory properties [16,160]. The oil has demonstrated effectiveness in treating infections and reducing inflammation, and has shown anticancer potential by inhibiting the growth of cancer cells in laboratory settings [161].

### 5.2. Agastache rugosa (Lamiaceae; Korean Mint)

*Agastache rugosa* is a perennial herb native to East Asia, particularly Korea, China, and Japan, and it is characterized by its aromatic, lance-shaped leaves and tall spikes of purple flowers [162]. The plant thrives in well-drained soils and is commonly cultivated for its medicinal and aromatic applications. The leaves release a pleasant, minty aroma when crushed, contributing to their popularity in herbal medicine and culinary applications. The essential oil of *Agastache rugosa* is rich in menthone, methyl chavicol (estragole), and linalool. Pharmacological studies show that the essential oil demonstrates diverse bioactivities [163], including antibacterial, antiasthematic, antifungal, antioxidant, and anti-inflammatory effects [164].

### 5.3. Abies koreana (Pinaceae)

*Abies koreana* is a coniferous tree native to the mountainous regions of Korea [165], recognized for its dense, aromatic foliage, and distinctive appearance, featuring short, dark green needles and a pyramidal shape. The tree typically grows at higher altitudes, thriving in cool, temperate climates [166]. The essential oil extracted from the needles and cones of *Abies koreana* is valued for its pleasant, refreshing aroma, often described as a blend of citrus and pine. This oil contains various bioactive compounds, including β-pinene, α-pinene, limonene, and camphene [165]. These compounds are primarily responsible for its characteristic fragrance, which has refreshing and calming effects. Pharmacological studies show that the essential oil of *Abies koreana* exhibits significant antioxidant, antimicrobial, and anti-inflammatory activities [167].

### 5.4. Artemisia spp. (Asteraceae)

*Artemisia* species—particularly *Artemisia annua*, *Artemisia japonica*, *Artemisia capillaris*, and *Artemisia iwayomogi*—are widely distributed across Korea and have long been used in traditional medicine [168]. These perennial or annual herbs thrive in temperate climates and are distinguished by their aromatic, feathery leaves and silvery-green foliage [169]. They typically produce small yellow or greenish-yellow clustered flowers, with essential oils responsible for their distinctive fragrance. These species are chemically diverse, rich in terpenoids, flavonoids, and phenolic acids [170]. *Artemisia annua* is particularly notable for producing artemisinin, a potent antimalaria compound that is a validated component of artemisinin-based combination therapies [171]. Additional bioactive compounds—such as camphor, borneol, eugenol, linalool, α-pinene, caryophyllene, eucalyptol, and thujone—contribute to their anti-inflammatory and antioxidant properties [140]. The essential oils of *Artemisia* spp. also exhibit antimicrobial, hepatoprotective, gastrointestinal, anti-inflammatory, and antioxidant effects [172].

### 5.5. Cinnamon spp. (Lauraceae)

*Cinnamomum loureirii* (Vietnamese cinnamon) and *Cinnamomum cassia* (cassia) are significant cinnamon species recognized in Korea for their aromatic essential oils and traditional medicinal uses [173]. These plants can grow up to approximately 10 m tall and are primarily cultivated for their bark, used in culinary and medicinal applications. Other cinnamon species, such as *Cinnamomum cassia*, also known as cassia, are commonly used in Korea [174]. The essential oils of *Cinnamomum loureirii* and *C. cassia* contain several bioactive compounds, with cinnamaldehyde being the primary contributor to their distinctive flavor and aroma [175]. Other significant constituents include eugenol, linalool, and coumarin [173]. Research shows that extracts of *Cinnamomum loureirii* inhibit acetylcholinesterase activity, indicating potential therapeutic application for cognitive disorders, such as Alzheimer’s disease. A study reveals that it ameliorates trimethyltin-induced cognitive dysfunction in mice, supporting its neuroprotective potential [175].

### 5.6. Citrus Species (Rutaceae)

Citrus species are widely cultivated in Korea for their fruits and medicinal properties. These include *Citrus sinensis* (sweet orange), *Citrus unshiu*, *Citrus reticulata* (mandarin), and *Citrus aurantium* (bitter orange), all characterized by glossy leaves, fragrant flowers, and juicy, tangy fruits [176]. Essential oils extracted from *Citrus* peels contain compounds such as limonene, linalool, and citral, which contribute to their distinct aroma and therapeutic benefits [177]. In Korea, *Citrus* species are valued for their anti-inflammatory, antioxidant, and antimicrobial properties [178]. They are used to improve digestion, relieve bloating, and reduce nausea due to their carminative effects. The peels are particularly rich in flavonoids, such as hesperidin and narirutin, which offer antioxidant protection against oxidative stress and inflammation [179]. Additionally, *Citrus* peel extracts and essential oils are incorporated into Korean cosmetics, where they help improve skin elasticity, reduce wrinkles, and protect against UV-induced damage [180].

### 5.7. Zanthoxylum ailanthoides (Rutaceae, Sichuan Pepper Tree)

*Zanthoxylum ailanthoides* is widely distributed in Korea and is known for its aromatic bark and leaves, which are often used in traditional Korean medicine [181]. It shares morphological and functional similarities with other species in the *Zanthoxylum* genus, such as *Zanthoxylum schinifolium* (Korean pepper tree) and *Zanthoxylum piperitum* (Japanese pepper tree), which are also valued for culinary and medicinal purposes [182]. The essential oils extracted from *Zanthoxylum ailanthoides* contain several bioactive compounds, including limonene, citronellal, and linalool [183]. These constituents contribute to the distinctive aroma and therapeutic properties of the plant, which include antibacterial, antiviral, and anti-inflammatory effects [184]. Limonene, in particular, is known for its strong citrus scent and its efficacy in managing various ailments, including digestive disorders, respiratory conditions, and inflammation [185].

### 5.8. Cryptomeria japonica (Cupressaceae, Japanese Cedar)

*Cryptomeria japonica* is native to Japan but is widely cultivated in Korea and other regions of East Asia. It is valued for its timber in construction and for its aromatic essential oils, which exhibit significant medicinal properties [186]. Essential oils extracted from the leaves and wood of *Cryptomeria japonica* possess a distinct aroma and contain various bioactive compounds, including monoterpenes such as α-pinene, β-pinene, and limonene, along with sesquiterpenes such as caryophyllene [186]. In Korea, *C. japonica* is used for its antimicrobial, antioxidant, anti-inflammatory, and insecticidal activities [187]. Research reveals that its essential oils possess strong antimicrobial effects, particularly against drug-resistant skin pathogens, supporting their use in treating infections [188].

### 5.9. Pinus spp. (Pinaceae, Korean Nut Pine)

Several *Pinus* species, including *Pinus densiflora* (Japanese red pine) and *Pinus koraiensis* (Korean nut pine), are commonly found in Korea and hold economic and medicinal significance [189]. These coniferous trees are characterized by their long, slender needles and produce cones containing seeds used for culinary and medicinal purposes [190]. The essential oils derived from *Pinus* species, particularly *Pinus densiflora* and *Pinus koraiensis*, contain bioactive compounds such as turpentine, α-pinene, β-pinene, and limonene [21]. These compounds contribute to the characteristic pine scent and are responsible for the pharmacological activities of the oils. The oils are known for their antimicrobial, antioxidant, anti-inflammatory, and insecticidal properties, making them valuable in traditional medicine and modern pharmaceutical applications [191].

### 5.10. Abies nephrolepis (Pinaceae, Korean Fir)

*Abies nephrolepis* is a coniferous tree native to Korea, distinguished by its dark green needles and conical form [192]. It primarily grows in mountainous regions, particularly around Seorak Mountain [193]. The essential oils extracted from *Abies nephrolepis* are rich in bioactive compounds, such as α-pinene, β-pinene, and camphene [194]. These oils possess antibacterial, antifungal, anti-inflammatory, and antioxidant effects, supporting their use in pharmaceutical and cosmetic formulations [195,196].

### 5.11. Thuja koraiensis (Cupressaceae)

*Thuja koraiensis* typically grows in mountainous regions of Korea and is valued for its ornamental appeal and medicinal properties [197]. The tree features dense evergreen foliage composed of small, scale-like leaves arranged in flat sprays, and it bears small, globose cones [198]. Essential oils from *Thuja koraiensis* are rich in compounds such as thujone, α-pinene, and β-pinene, which contribute to its distinctive fragrance and various therapeutic properties [199]. These oils exhibit antimicrobial, antiviral, anti-inflammatory, and anticancer effects. They show significant efficacy against pathogens affecting the respiratory and gastrointestinal systems [200]. Studies reveal that *Thuja koraiensis* extract exhibits potent antibacterial and antiviral effects, supporting its potential use in infection treatment [201].

### 5.12. Magnolia kobus (Kobus Magnolia; Magnoliaceae; Moknyeon)

*Magnolia kobus* is a deciduous tree native to Korea and Japan, commonly found in temperate forests. It is characterized by its striking white flowers, which bloom in early spring, and has a moderate growth rate, reaching heights of up to 12 m [202]. Due to its aesthetic appeal, it is widely cultivated in gardens and used as an ornamental tree [203]. The essential oils extracted from *Magnolia kobus* are rich in bioactive compounds, including caryophyllene, α-terpineol, and other terpenoids [204]. These oils contribute to its therapeutic properties, including anti-inflammatory effects (through the suppression of NF-κB and activation of Nrf2 signaling pathways), antioxidant properties, and antimicrobial effects [205]. Studies reveal that flower buds of *Magnolia kobus*, particularly those extracted using supercritical carbon dioxide, exhibit significant pharmacological activities. These include enhancement of brain function and concentration through modulation of electroencephalographic patterns [206].

### 5.13. Zingiber officinale (Korean Bongdong (Bg) Cultivar; Zingiberaceae)

**Figure 5:**
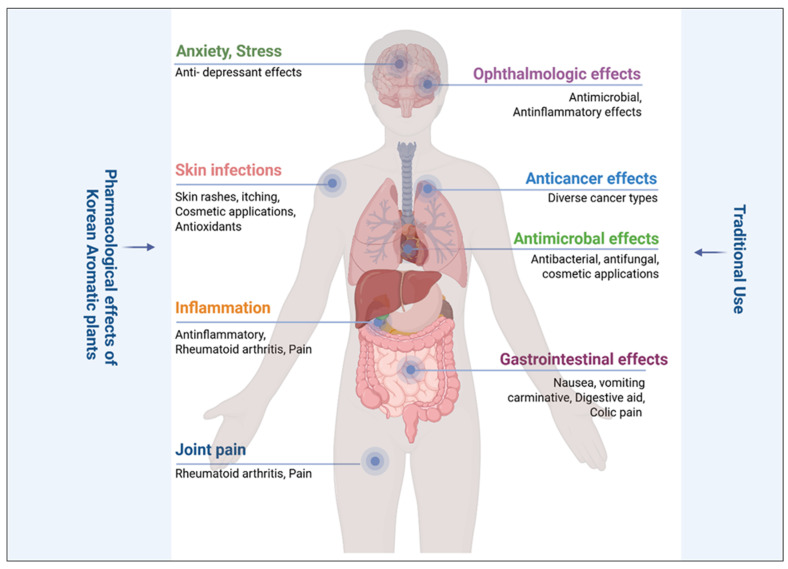
Overview of pharmacological properties of Korean aromatic plants.

The Bondong cultivar of *Zingiber officinale* is indigenous to Korea and has been cultivated for a long time in the “Bongdong” region in Korea [207]. It has been reported to posess limonene, zingiberene, geranial, camphene, neral, and curcumene + geranyl acetate in the rhizome part [208]. The volatile profile of etiolated shoots (sprouts) differed significantly, showing a higher concentration of terpene hydrocarbons and lower levels of citral [209]. Extracts of Korean ginger (including ethanol and ethyl acetate fractions) showed high antioxidant activity, efficacy in lowering blood pressure, and carminative, antiulcer effects [210,211]. A detailed overview of the pharmacological effect of Korean aromatic plants is shown in Figure 5.

**Table 3:** Common Korean aromatic plants and their detailed pharmacological profiles.

S. No	Species Name	Major Chemical Components	Pharmacological Activities	Investigation Type	Citation
1	*Pinus thunbergii* Parl.	α-pinene, β-pinene, and bornyl acetate	Antioxidant, anti-inflammatory, and antimicrobial	*In vitro*	[80]
2	*Abies koreana* E.H. Wilson	α-pinene, limonene, and camphene	Anti-inflammatory, antioxidant, and antimicrobial	*In vitro*	[212]
3	*Machilus japonica* Siebold and Zucc.	Lignans (syringaresinol and secoisolariciresinol) and terpenoids	Antioxidant, anticancer, and anti-inflammatory	*In vitro*	[213]
4	*Cinnamomum camphora* (L.) J. Presl	Camphor, eugenol, and cinnamaldehyde	Antioxidant, antimicrobial, and anti-inflammatory	*In vitro*	[90]
5	*Cinnamomum loureirii* Nees	Linalool, eugenol, and cinnamaldehyde	Anti-inflammatory, antioxidant, and anticancer	*In vitro*	[90]
6	Neolitsea sericea (Blume) Koidz.	β-caryophyllene, α-pinene, and eucalyptol	Antioxidant and anti-inflammatory	*In vitro*	[91]
7	*Zanthoxylum ailanthoides* Siebold and Zucc.	Bergamottin, piperine, and limonene	Antioxidant, antimicrobial, and anti-inflammatory	*In vitro*	[15]
8	*Citrus reticulata* Blanco	Limonene, β-pinene, and α-terpinene	Antioxidant, anticancer, and anti-inflammatory	*In vitro*	[92]
9	*Citrus unshiu* (Yu. Tanaka ex Swingle) Marcow.	Limonene, γ-terpinene, and α-pinene	Anti-inflammatory, antioxidant, and antimicrobial	*In vitro*	[15]
10	*Zanthoxylum coreanum* Nakai	Bergamottin, limonene, and piperine	Antioxidant and anti-inflammatory	*In vitro*	[15]
11	*Cryptomeria japonica* (Thunb. ex L.f.) D. Don	Lignans (cryptomeridiol and α-terpineol) and flavonoids	Anti-inflammatory, antioxidant, and anticancer	*In vitro*	[214]
13	*Magnolia kobus* DC.	Magnolol, honokiol, and eugenol	Anti-inflammatory, antioxidant, and anticancer	*In vitro*	[99]
14	*Vitex rotundifolia* L.f.	Casticin, vitexin, and apigenin	Antioxidant, anti-inflammatory, and antimicrobial	*In vitro*	[15]
15	*Chamaecyparis pisifera* (Siebold and Zucc.) Endl.	Isofraxidin, lignans, and α-pinene	Antimicrobial, anti-inflammatory, and antioxidant	*In vitro*	[15]
16	*Artemisia hallaisanensis*	Flavonoids (rutin and quercetin) and terpenoids (camphor and β-caryophyllene)	Anti-inflammatory, antioxidant, and antimicrobial	*In vivo*	[215]
17	*Artemisia japonica* ssp. *littoricola*	α-pinene, β-caryophyllene, and artemisinin	Anti-inflammatory, antioxidant, and antimicrobial	*In vivo*	[215]
18	*Pinus rigida* Mill	α-pinene, β-pinene, and bornyl acetate	Antioxidant, anti-inflammatory, and antimicrobial	*In vitro*	[80]
19	*Pinus densiflora* for. *multicaulis*	α-pinene, β-pinene, and bornyl acetate	Antioxidant, anti-inflammatory, and antimicrobial	*In vitro*	[80]
20	*Pinus parviflora* Siebold & Zucc.	α-pinene, β-pinene, and bornyl acetate	Antioxidant, anti-inflammatory, and antimicrobial	*In vitro*	[80]
21	*Agastache rugosa* (Fisch. and CA Mey.) Kuntze	Rosmarinic acid, luteolin, and apigenin	Antimicrobial, anti-inflammatory, and antioxidant	*In vitro*	[91]
22	*Magnolia kobus* DC.	Magnolol, honokiol, and eugenol	Anti-inflammatory, antioxidant, and anticancer	*In vitro*	[15]
23	*Pinus densiflora* for. multicaulis	α-pinene, β-pinene, and bornyl acetate	Antioxidant, anti-inflammatory, and antimicrobial	*In vitro*	[80]
24	*Abies nephrolepis* (Trautv. ex Maxim.) Maxim.	α-pinene, camphene, and terpinolene	Anti-inflammatory, antioxidant, and antimicrobial	*In vitro*	[100]
25	*Pinus parviflora* Siebold and Zucc.	α-pinene, β-pinene, and bornyl acetate	Antioxidant, anti-inflammatory, and antimicrobial	*In vitro*	[80]
27	*Pinus koraiensis* Siebold and Zucc.	α-pinene, β-pinene, and bornyl acetate	Antioxidant, anti-inflammatory, and antimicrobial	*In vitro*	[80]
28	*Thuja koraiensis* Nakai	Lignans (tanoic acid) and phenolic acids	Antimicrobial, anti-inflammatory, and anticancer	*In vitro; In vivo*	[198]
30	*Juniperus chinensis* L.	α-pinene, β-pinene, and sabinene	Antioxidant, anti-inflammatory, and antimicrobial	*In vitro*	[91]

## 6. Molecular Phylogenetics

Molecular phylogenetics has significantly advanced the understanding of plant evolution by elucidating evolutionary relationships, divergence times, and speciation patterns [216]. Many morphologically similar but genetically distinct species benefit greatly from DNA-based analyses, which complement traditional taxonomy and chemotaxonomy [86]. The application of nuclear and plastid molecular markers establishes a nice frameworks for reconstructing phylogenies and resolving taxonomic ambiguities among endemic taxa [217]. Advances in high-throughput sequencing and bioinformatics tools have further enabled comprehensive genomic analyses, allowing for higher-resolution insights into lineage divergence and ancestral trait evolution.

### 6.1. Molecular Markers in Phylogenetic Studies

Molecular markers are essential tools in plant phylogenetic studies, providing genetic-level insights into evolutionary relationships among species and populations [217]. By detecting genetic variation within and between taxa, these markers have transformed the study of plant evolution, enabling the tracing of lineage relationships and the elucidation of processes such as speciation, hybridization, and adaptation [218,219]. Their application has significantly enhanced the resolution and accuracy of phylogenetic trees, deepening our understanding of plant biodiversity and evolutionary dynamics [220]. Plant molecular markers are broadly categorized into two primary types: nuclear and plastid markers [221]. Nuclear markers, which are inherited biparentally, are particularly informative for examining genetic diversity and evolutionary history at the species and genus levels [222]. Commonly used nuclear markers include microsatellites, ribosomal DNA, and single-nucleotide polymorphisms (SNPs) [223,224]. These markers detect genetic variation across various taxonomic levels from individual species to broader taxonomic groups—and provide valuable insights into population structure, gene flow, and genetic differentiation [225].

In addition to nuclear markers, plastid markers, which are maternally inherited in most plants, are frequently utilized in plant phylogenetic studies [226]. Chloroplast DNA markers, including regions such as *rbcL*, *matK*, and *trnL-F*, are widely used to assess phylogenetic relationships within and between families, genera, and species [227]. The maternal inheritance of plastid DNA eliminates the confounding effects of recombination, making these markers ideal for exploring deeper evolutionary lineages [228]. Furthermore, plastid markers exhibit lower mutation rates than those of nuclear markers, which makes them highly effective for resolving evolutionary patterns at the species and genus levels [229]. Mitochondrial DNA markers are also gaining attention in plant phylogenetics, although their application remains more limited than that of nuclear and plastid markers [230]. Plant mitochondrial genomes tend to evolve slowly but often show significant variability among closely related species. Mitochondrial markers, such as the cytochrome c oxidase subunit I gene, are valuable for investigating deeper evolutionary histories and studying organelle evolution and inheritance patterns [231]. However, the use of mitochondrial markers in plants is less common due to their complex inheritance patterns and limited variability in some plant taxa [232].

The advent of next-generation sequencing (NGS) technologies has significantly enhanced the application of molecular markers in plant phylogenetic research [233]. With the ability to sequence large quantities of DNA at relatively low cost, NGS has enabled the simultaneous analysis of numerous genetic loci, thereby enhancing phylogenetic resolution and deepening our understanding of plant evolutionary history [234]. NGS platforms support the sequencing of entire genomes or targeted genomic regions using approaches such as target capture or amplicon sequencing, allowing for the exploration of complex plant phylogenies with high precision. Integrating NGS with molecular marker data provides a more comprehensive approach to plant phylogenetics, offering insights into genetic diversity, speciation, and the evolutionary mechanisms underlying plant biodiversity [233].

Recent studies applying multilocus datasets and concatenated analyses reveal greater resolution within taxonomically challenging plant groups. Advancements in NGS technologies, such as genome skimming and target capture, have allowed the acquisition of large datasets even from herbarium specimens, facilitating phylogenetic reconstruction at unprecedented scales [235]. These methods enable the identification of SNPs and insertion–deletion mutations, which are essential for resolving complex species relationships.

### 6.2. Phylogenetic Relationships and Evolutionary History

Several researchers have actively investigated the phylogenetic relationships of Korean aromatic plants, including endemic plants. Among these, the complete chloroplast (cp) genome sequence of Korean endemic aromatic plant *Thymus quinquecostatus* var. *japonicus*, traditionally used in folk remedies, was analyzed for the first time in this study. The cp showed a typical circular structure measuring 151,782 bp in length, comprising a large single-copy region (82,903 bp), a small single-copy region (17,667 bp), and two inverted repeat regions (25,606 bp). Phylogenetic analysis using cp genome sequences confirms that *Thymus quinquecostatus* var. *japonicus* is a sister taxon to members of the genus *Mentha* within the subfamily Nepetoideae [236].

*Agastache rugosa* (Korean mint), an aromatic perennial plant from the Lamiaceae family, is recognized for its medicinal properties and antioxidant compounds. To enhance its agronomic traits and secondary metabolite biosynthesis, a chromosome-level genome was constructed using Nanopore sequencing and Hi-C technology. The final assembly spanned 410.67 Mbp and comprised nine pseudochromosomes, representing 89.1% of the total genome. The assembly exhibited high completeness, with 561,061 repeat elements and 26,430 predicted protein-coding genes. This chromosome-scale genome provides a valuable resource for understanding the genetic architecture of *A. rugosa* and advancing its industrial applications [237].

Furthermore, Korean endemic *Artemisia* species, including *Artemisia keiskeana* and *Artemisia takeshimensis*, have undergone multilocus phylogenetic analysis using plastid genes and nuclear markers. These analyses confirmed their monophyly and evolutionary divergence from related taxa in Northeast Asia. The chloroplast genomes of 18 *Artemisia* taxa from Korea revealed a conserved structure containing 87 protein-coding genes, 37 tRNAs, and 8 rRNAs. Evolutionary analysis showed variation in the *trnH-psbA* intergenic spacer and signs of selection in the *accD* and *ycf1* genes. Phylogenetic analysis clustered the plastomes into five distinct groups, supporting a monophyletic subgenus *Dracunculus* and a paraphyletic subgenus *Artemisia*. These plastomes serve as molecular markers for identifying chloroplast haplotypes, while the *accD* and *ycf1* hotspots provide highly informative sites for developing discriminatory markers within the Asteraceae family. These findings establish *Artemisia* plastomes as super-barcodes for future phylogenomic studies [238].

Similarly, sweet marjoram (*Origanum majorana*), an aromatic herb in the Lamiaceae family, was analyzed for its complete chloroplast nucleotide sequence and phylogenetic relationship with other Lamiaceae species. The plastome was 151,841 bp long, comprising a pair of inverted repeats (25,558 bp each), a large single-copy region (83,035 bp) and a small single-copy region (17,690 bp). The genome encodes 132 genes, including 86 protein-coding genes, 36 tRNA genes, and 8 rRNA genes. Phylogenetic analysis indicates that *O. majorana* is most closely related to *Origanum vulgare* [239].

*Abies koreana*, an endangered conifer endemic to the high-altitude regions of South Korea, is highly susceptible to climate change. To elucidate its complex evolutionary history shaped by hybridization and reticulate evolution, the complete mitochondrial (1,174,803 bp) and plastid (121,341 bp) genomes were sequenced and assembled. The mitochondrial genome exhibits significant structural dynamics, including cis-to-trans splicing transitions and disruption of conserved gene clusters. In contrast, the plastome presents two distinct structural conformations associated with short, inverted repeats (1186 bp), as revealed by ONT reads. Transcriptome analysis was used to identify 1356 C-to-U RNA editing sites across 41 mitochondrial genes. These findings highlight the phylogenomic complexity within Abies, providing strong support for sectional relationships, and emphasize the value of integrating multiple genomic compartments to resolve reticulate evolution in conifers [240].

Similarly, yuzu (*Citrus junos* Sieb. Ex Tanaka), a major *Citrus* species cultivated in China, Japan, and Korea, is presumed to be a natural hybrid. However, definitive evidence confirming its hybrid origin has not been reported. To identify putative maternal species, nucleotide sequences of five hypervariable regions totaling 17,531 bp in citrus chloroplast genomes were obtained from yuzu. A phylogenetic tree based on these sequences shows that yuzu is most closely related to Ichang papeda (*C. cavaleriei*), suggesting that Ichang papeda may be the seed parent of yuzu. To determine the paternal species, yuzu homologs of 103 single-copy genes present in citrus genomes were identified from a yuzu transcriptome that was de novo assembled using RNA-seq data. Eighty homologous genes were retrieved from the yuzu transcriptome. A phylogenetic tree based on nucleotide sequence variations in these 80 single-copy genes across *Citrus* species shows that yuzu is a hybrid between Ichang papeda and mandarin [241].

Among plants in the *Pinaceae* family, the genome of *Populus koreana* was sequenced and assembled into 19 chromosomes, comprising a total size of 429.47 Mb and 38,075 predicted genes. Phylogenetic analysis suggests that *P. koreana* and *Populus trichocarpa* diverged approximately 6.14 million years ago. Metabolomic analysis reveals that terpenes and esters, key components of aroma, are significantly enriched in *P. koreana* leaves. The *P. koreana* genome contains more terpene synthase (TPS) genes than *P. trichocarpa*, particularly TPS-a/b genes, which are likely involved in sesquiterpene and monoterpene biosynthesis. The expansion of gene families related to terpenoid biosynthesis in *P. koreana*, primarily through tandem and whole-genome duplications, provides valuable insights into the evolution of plant volatiles. This genomic information serves as a valuable genomic resource for breeding and investigating aromatic traits in forest trees [242]. Table 4 shows the details of other investigations. These findings reveal that these species diverged from their continental relatives due to geographic isolation and niche differentiation in the montane environments of Korea.

### 6.3. Integrative Approaches Combining Molecular Phylogenetics and Chemotaxonomy

Integrative approaches that combine molecular phylogenetics and chemotaxonomy have become central to modern plant systematics, facilitating a more accurate and comprehensive understanding of plant relationships [246]. These methods merge molecular genetic data with the study of secondary metabolites to enhance taxonomic resolution and elucidate evolutionary relationships. By incorporating molecular phylogenetics, which examines genetic-level evolutionary changes, with chemotaxonomic markers such as alkaloids and terpenoids, researchers can differentiate closely related species and explore their biochemical diversity [247].

A significant example is the application of integrated molecular and chemotaxonomic analyses to the genus *Rubia*. Researchers combined molecular phylogeny with chemotaxonomy to explore the origin and distribution of *Rubia* species, which are characterized by unique chemotypic profiles, including anthraquinone derivatives, that aid in species authentication. The genus *Rubia* (Rubiaceae), recognized for its chemical diversity—including quinones, cyclopeptides, and triterpenoids has gained attention for its potential anti-tumor properties. However, species identification remains challenging due to morphological similarities among taxa. To address this, an integrative strategy was employed in this study, involving morphological traits, molecular phylogenetics, and chemotaxonomic profiling. DNA barcoding using chloroplast markers and the internal transcribed spacer region revealed distinct clades, although relationships within *sect. Oligoneura* remains unresolved. Non-targeted metabolomic analysis was used to identify triterpenes and Rubiaceae-type cyclopeptides (RAs) as key chemotaxonomic markers. The study also suggests that Southwest China serves as the center of origin and a biodiversity hotspot for *Rubia* species. Overall, the findings underscore the value of integrating molecular and chemical approaches for accurate plant authentication and for optimizing high-quality germplasm for RA production [248].

However, Korean aromatic plants have also been explored in the context of molecular phylogenetics and chemotaxonomy. In a study on the chemotaxonomic classification of *Peucedanum japonicum*, a Korean medicinal aromatic plant, researchers integrated molecular phylogenetic analysis with chemical profiling to distinguish it from related species such as *Peucedanum praeruptorum* and *Angelica decursiva*. This combined approach not only clarified evolutionary relationships but also revealed specific chemical markers, providing deeper insights into the phylogeny and taxonomy of these species [249]. Another significant example is the study of Korean *Chrysanthemum*, an iconic native aromatic plant found in Korea. This research employed morphological, molecular, and chemotaxonomic data to resolve the taxonomic status of various *Chrysanthemum* species. By analyzing genetic markers and phytochemicals, this study provided a comprehensive understanding of species boundaries and regional variation [250].

Similarly, a detailed investigation was conducted on the diversity of secondary metabolites in relation to genetic variation across nine Lauraceae species, including *C. camphora*, *C. yabunikkei*, *L. erythrocarpa*, *L. coreana*, *L. japonica*, *M. japonica*, *M. thunbergi*, *N. aciculate*, and *M. sericea.* Plant samples were chemically analyzed and classified. Multivariate statistical methods, such as PLS-DA, were applied to identify key metabolites responsible for distinguishing the nine species. These metabolites were further characterized using preparative LC-MS and MS/MS fragment pattern analysis. In addition, the chemical dendrogram generated through molecular network analysis was compared with that of the genetic dendrogram. This integrative approach enabled a comprehensive comparison of the chemical profiles across multiple species, thereby enhancing the understanding of phylogenetic relationships within Lauraceae [251]. Additionally, the integration of untargeted metabolomics, DNA marker-based sequencing, and bio-imaging represents an innovative direction in plant integrative taxonomy. This strategy facilitates the identification of chemical profiles that reflect evolutionary divergence and ecological adaptations, providing deeper insights into plant systematics [252].

## 7. Conservation and Sustainable Utilization

The conservation and sustainable utilization of Korean aromatic plants are essential for preserving their genetic diversity and ecological integrity. Many of these species are confined to specialized and vulnerable habitats, such as mountainous regions, coastal cliffs, and isolated islands. They face growing threats from habitat loss, overexploitation, invasive species, and climate change [15]. Rapid urbanization, agricultural expansion, and tourism development in Korea have led to the fragmentation and degradation of natural habitats. These disturbances have significantly affected the population dynamics of numerous endemic aromatic plants [44,253]. Furthermore, increasing commercial demand for aromatic plant products has intensified harvesting pressures, often exceeding sustainable levels and placing wild populations at risk. In this context, effective conservation strategies are needed to integrate scientific research, policy development, and community engagement. Such integrated approaches are essential for protecting these species while promoting their sustainable use for economic and medicinal applications.

### Conservation Status of Korean Aromatic Plants

Comprehensive assessments of the conservation status of Korean endemic aromatic plants indicate that many are categorized as vulnerable, endangered, or critically endangered under national Red Lists and the International Union for Conservation of Nature criteria [12,38]. These assessments consider factors such as restricted geographic distribution, population decline, and habitat degradation. For example, endemic species inhabiting the Baekdudaegan mountain range and Jeju Island exhibit heightened sensitivity to environmental disturbances and climate-induced changes in habitat suitability [254]. Genetic studies indicate that reduced genetic diversity in fragmented populations increases their susceptibility to stochastic events and limits their adaptive capacity [255]. Additionally, climate change projections suggest upward shifts in suitable habitat ranges, which may lead to habitat loss for species confined to mountaintops or island ecosystems, thereby intensifying extinction risks [256].

Effective conservation assessments increasingly utilize integrative approaches that combine field surveys, population genetics, and ecological niche modeling [257]. These methods help identify priority species and habitats for protection by pinpointing vulnerability hotspots and evolutionarily significant units. Ex situ strategies, such as seed banking and living collections in botanical gardens, complement in situ conservation by preserving genetic resources for future restoration and research [258]. However, the success of these initiatives depends on regular monitoring and timely updates to conservation status, ensuring responsiveness to emerging threats and shifting population dynamics (Table S2).

## 8. Challenges and Limitations

Integrating molecular phylogeny and chemotaxonomy provides significant insights into plant systematics, but it also presents numerous challenges and limitations. A key difficulty lies in reconciling data from distinct scientific domains [259]. Molecular phylogeny relies on genetic information, such as DNA sequences, while chemotaxonomy focuses on secondary metabolites, which may vary within species due to environmental or developmental influences [260]. This variability can complicate species comparisons and reduce the reliability of chemotaxonomic markers. Another limitation lies in the difficulty of obtaining comprehensive datasets for all species [248]. Molecular analyses are often constrained by incomplete or unavailable genomic data, particularly for rare or understudied taxa [261]. Similarly, chemotaxonomic studies require detailed chemical profiles, which may be restricted by the high cost and technical demands of analytical techniques such as chromatography and mass spectrometry [109].

**Figure 6:**
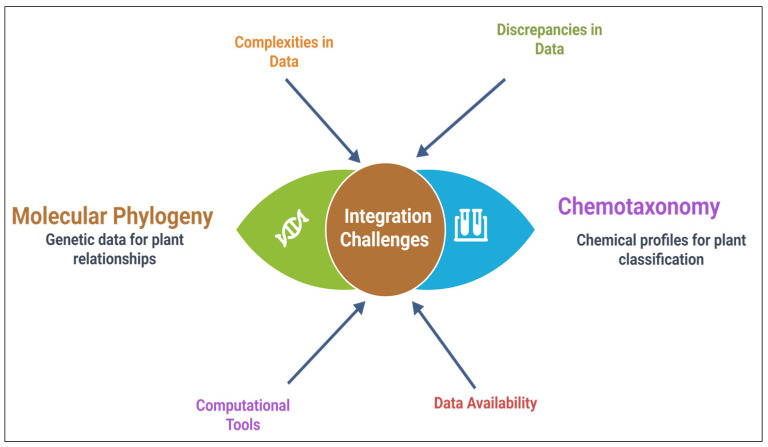
Challenges in integrating molecular phylogenetic analysis with chemotaxonomic evaluation of aromatic plants.

In addition, integrating molecular and chemical data presents computational challenges [262]. While molecular data can be analyzed using standard bioinformatics pipelines, chemical data often requires specialized metabolomic software. Combining these datasets often demands advanced interdisciplinary expertise [263]. Although integrative approaches are promising, they are often limited by the assumption that molecular and chemical data will provide clear, complementary insights. In reality, discrepancies between molecular phylogeny and chemotaxonomy are common. These inconsistencies may result from horizontal gene transfer, convergent evolution, or plasticity in secondary metabolite production. Such factors complicate the resolution of taxonomic relationships [151]. In the context of aromatic plants from the Republic of Korea, such discrepancies have been particularly apparent. For instance, species within the *Artemisia* genus are widely distributed and used in traditional Korean medicine, exhibit high variability in composition of essential oils even within morphologically similar species. While molecular phylogeny places certain species in closely related clades, chemotaxonomic profiles (especially based on terpenoid content) often suggest divergent groupings. This is more likely due to ecological adaptation or seasonal effects on secondary metabolite production. (Figure 6).

## 9. Strategies for Conservation and Sustainable Use

Sustainable conservation of Korean aromatic plants requires a multifaceted approach that integrates in situ and ex situ strategies tailored to species-specific ecological needs [264]. In situ conservation focuses on protecting natural habitats through the creation and management of protected areas, including national parks and nature reserves, particularly within biodiversity hotspots such as the Baekdudaegan range and Jeju Island [265]. Habitat restoration and invasive species control are essential components of these efforts, enhancing ecosystem resilience and facilitating natural regeneration. Additionally, conservation corridors and landscape-level planning are employed to reduce habitat fragmentation and facilitate gene flow among isolated populations [266].

Ex situ conservation focuses on propagating and cultivating endemic aromatic plants under controlled conditions to reduce pressure on wild populations and ensure sustainable supplies for research and commercial applications [267]. Advances in tissue culture and micropropagation techniques enable the rapid multiplication of rare species while preserving genetic integrity [268]. Cultivation practices optimized for secondary metabolite production can enhance yields of bioactive compounds, supporting pharmaceutical and cosmetic industries. Furthermore, community-based conservation initiatives that involve local stakeholders in sustainable harvesting, benefit-sharing, and traditional knowledge preservation promote stewardship and long-term conservation success.

Policy frameworks and regulatory mechanisms are also crucial for effective conservation and sustainable use [269]. The enforcement of harvesting quotas, trade restrictions, and certification standards for sustainably sourced products helps prevent overexploitation of resources. Incorporating scientific evidence into policymaking facilitates adaptive management in response to evolving environmental and socioeconomic conditions. International cooperation and adherence to global agreements, such as the Convention on Biological Diversity, further strengthen conservation outcomes [270].

## 10. Conclusions

In conclusion, Korean endemic aromatic plants constitute a unique and valuable component of the biodiversity found on the Korean peninsula, characterized by distinctive chemical compositions, complex phylogenetic relationships, and diverse biological activities. The integration of chemotaxonomic, molecular phylogenetic and bioactivity data has significantly enhanced our understanding of their taxonomy, evolutionary trajectories, and therapeutic potential. These species hold ecological and evolutionary significance, offering promising prospects for the discovery and sustainable utilization of natural products. However, their restricted distribution, habitat specificity, and increasing anthropogenic pressures have rendered many species vulnerable, highlighting the need for urgent, coordinated conservation strategies supported by extensive scientific research. Despite considerable progress, several challenges remain in fully characterizing and utilizing these plants. Taxonomic uncertainties, often arising from cryptic species and hybridization, require continued refinement through integrative approaches that combine genomics, metabolomics, and advanced bioinformatics. The complexity of secondary metabolite biosynthesis, coupled with environmental influences on chemical expression, complicates chemotaxonomic classifications and bioactivity assessments, necessitating standardized protocols and expanded metabolomic datasets. Conservation efforts are further constrained by habitat degradation, climate change, and inadequate ex situ propagation techniques, all of which threaten the genetic diversity and long-term availability of these plants. Future research should prioritize high-throughput sequencing and multi-omics integration to resolve phylogenetic relationships and identify biosynthetic gene clusters responsible for bioactive compound production. Advances in metabolomics and machine learning hold promise for predictive chemotaxonomy and accelerated discovery of bioactive molecules. Overall, overcoming these challenges will require multidisciplinary collaboration and supportive policies to unlock the full scientific and socio-economic potential of the aromatic flora found in Korea.

## Figures and Tables

**Table 1:** Keywords and search results for the systematic literature review adopted during the investigation.

Search String	Database	Articles Found
TITLE-ABS-KEY (“Korean aromatic plants” OR “Essential oil containing plants in Korea” OR “aromatic plants from Korean peninsula”) AND (“aromatic plant *” OR “medicinal plant *” OR “aromatic herbs *” OR “Aromatic flowers *” OR “aromatic leaves *” OR “Common Korean Aromatic herbs *”)	PubMed	16
	Web of Science	21
	Scopus	28
TITLE-ABS-KEY (“Korean aromatic plants hotspots” OR “Korean aromatic plants in islands” OR “Korean aromatic plants in mountain range”) AND (“Korean Aromatic plants occurrence *” OR “Korean Aromatic plants prevalence *” OR “distribution of Aromatic plants across South Korea *”)	PubMed	14
	Web of Science	22
	Scopus	31
TITLE-ABS-KEY (“Chemistry of Korean aromatic plants” OR “Essential oil composition of in Korean plants” OR “Phytochemistry of Korean aromatic plants”) AND (“Chemotaxonomy of Korean Aromatic plants *” OR “Chemotaxonomically evaluation of Korean Aromatic plants *” OR “GC-Ms analysis of Korean Aromatic plants *” OR “Chemotaxonomical evaluation of Korean fragrant plants *” OR “aromatic leaves chemotaxonomy *”)	PubMed	16
	Web of Science	19
	Scopus	35
TITLE-ABS-KEY (“Molecular phylogenetic analysis” OR “Phylogenetic analysis” OR “Molecular phylogeny” OR “Molecular systematics”) AND (“Chemotaxonomy” OR “Taxonomy” OR “Plant taxonomy” OR “Phylogenetic relationships” OR “Evolutionary relationships”) AND (“Molecular markers” OR “Genetic markers” OR “DNA barcoding” OR “rDNA sequencing” OR “Next-generation sequencing” OR “Genetic analysis *”)	PubMed	12
	Web of Science	18
	Scopus	35
TITLE-ABS-KEY (“Integrated plant taxonomy” OR “Plant taxonomy” OR “Plant systematics” OR “Taxonomic integration” OR “Taxonomic classification”) AND (“Plant classification” OR “Phylogenetic relationships” OR “Evolutionary relationships” OR “Molecular taxonomy” OR “Molecular systematics”) AND (“Genetic markers” OR “Molecular markers” OR “DNA barcoding” OR “Next-generation sequencing” OR “rDNA sequencing” OR “Genetic analysis”) AND (“Ecological relationships” OR “Plant biodiversity” OR “Plant identification” OR “Phylogenetic analysis *”)	PubMed	16
	Web of Science	20
	Scopus	39
TITLE-ABS-KEY (“Pharmacology” OR “Pharmacological properties of Korean aromatic plants” OR “Pharmacological properties of Korean aromatic plants” OR “Pharmacological evaluation of Korean aromatic plants” OR “Phytopharmacology”) AND (“Traditional uses of Korean aromatic plants” OR “Traditional aromatic Korean medicine” OR “Ethnopharmacology of Korean aromatic plants” OR “Herbal medicine” OR “Traditional healing of Korean aromatic plants”) AND (“Antioxidant Korean Aromatic plants” OR “Anti-inflammatory Korean aromatic plants” OR “Antibacterial Korean aromatic plants” OR “Anticancer Korean aromatic plants” OR “Antifungal Korean aromatic plants” OR “Antiviral Korean aromatic plants” OR “Immunomodulatory Korean aromatic plants”)	PubMed	16
	Web of Science	20
	Scopus	42
Sum		420

**Table 4:** Phylogenetic studies of Korean aromatic Plants using molecular markers.

S. No.	Plant Name	Genetic Markers Used	Phylogenetic Analysis Findings	Citation
1	*Thymus quinquecostatus*	Complete Chloroplast genome	Clustered with other Lamiaceae species, indicating close evolutionary relationships within the genus *Thymus*.	[236]
2	*Agastache rugosa*	entire protein-coding gene set	Closely related to other *Agastache* species, showing high genetic similarity within the Lamiaceae family.	[229]
3	*Artemisia* spp.	Whole plastome sequences, *trnH–psbA*, *accD*, *and ycf1*	Nested within the *Artemisia* genus, sharing high sequence similarity with other Asteraceae members.	[238]
4	* Origanum majorana *	Complete chloroplast genome	Phylogenetic analysis confirmed its closest relationship to *Origanum vulgare*.	[231]
5	*Abies koreana*	rDNA cluster, 452 single-copy genes, tRNAs, and rRNAs	Positioned within the Pinaceae family, closely related to other Abies species, indicating a shared ancestor in temperate conifers.	[240]
6	* Citrus junos *	*Chlororplast matK*, *rps16*, *rpoC1*, *rpoB*, *psbD; and nuclear* 80 single-copy genes	Confirmed to be a hybrid, likely derived from *Ichang papeda* and mandarin as parental species.	[241]
7	*Populus koreana*	RAD-seq	Restriction site-associated DNA sequencing (RAD-seq)	[242]
8	*Abies nephrolepis*	Nuclear microsatellite markers, as well as mitochondrial and chloroplast DNA markers	Provided insights into evolutionary processes and genetic diversity in *Abies nephrolepis* populations in Northeast Asia.	[192]
9	* Thuja koraiensis *	Genome dataset (242 SNPs)	Revealed geographically random patterns of genetic variation.	[243]
10	*Tsuga sieboldii*	cpSSRs,	Genome sizes and gene contents were relatively conserved among the species, reflecting structural stability within the genus.	[244]
11	*Zanthoxylum schinifolium*	SSR and IR	Enhanced understanding of genetic diversity and evolutionary relationships within the Zanthoxylum genus.	[245]

rDNA, ribosomal DNA; SNPs, single-nucleotide polymorphisms; RAD-seq, restriction site-associated DNA sequencing.

## Data Availability

No new data were created or analyzed in this study.

## Supplementary Materials

The following supporting information can be downloaded at: https://www.mdpi.com/article/10.3390/plants14152364/s1, Figure S1: Chemical structure of major phytochemicals identified in essential oils; Table S1: A comparative of Chemotaxonomical analysis with earlier reviews. Table S2: Summary of Bioactivity Studies and Knowledge Gaps in Korean Aromatic Plants.
