# Supplementary material for: Harnessing Molecular Phylogeny and Chemometrics for Taxonomic Validation of Korean Aromatic Plants: Integrating Genomics with Practical Applications
Source: Plants (Basel). 2025 Aug 1;14(15):2364. doi: 10.3390/plants14152364 (PMC12348784; doi:10.3390/plants14152364)
Supplement: Supplementary file 1 [file plants-14-02364-s001.zip › plants-3758307-supplementary.pdf]

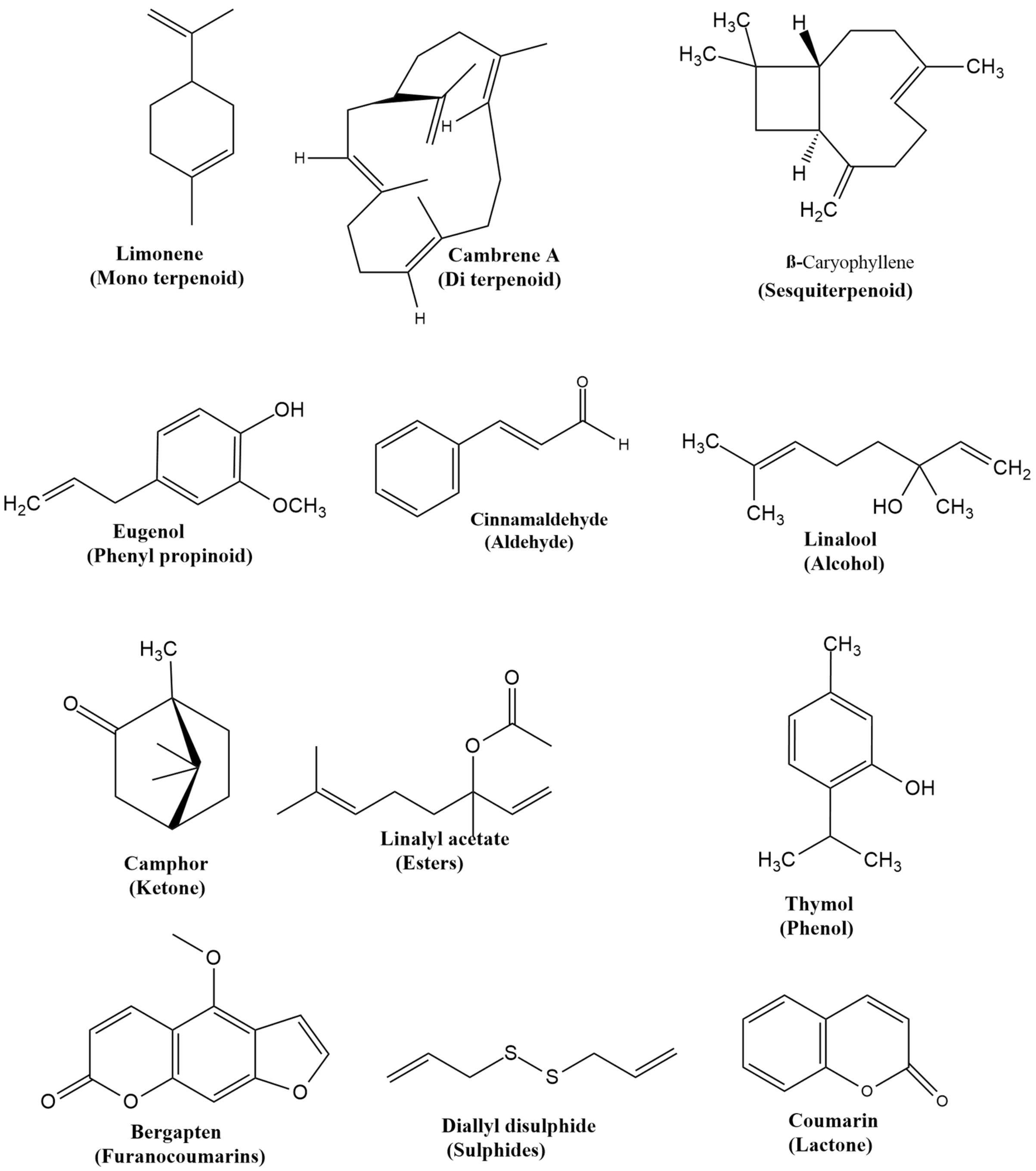

Figure S1. Chemical structure of major phytochemicals identified in essential oils.

Table S1: A comparative of Chemotaxonomical analysis with earlier reviews.

Aspect	This Review	Mali et al. 2023 [1]	Ramos et al. 2023[2]
**Focus of Review**	Chemotaxonomy of Korean aromatic plants	Global chemotaxonomy and chemophenetics	Chemotaxonomy and chemodiversity in plants
**Phytochemical Markers Examined**	Essential oils, terpenoids, phenolics, alkaloids, polysaccharides	Alkaloids, flavonoids, terpenoids	Secondary metabolites, chemodiversity, chemophenetics
**Methodologies Used**	Chromatography, NMR, GC-MS, metabolomics	Chromatography, GC-MS, and HPLC	GC-MS, LC-MS, HPLC, metabolomics
**Geographic Focus**	Korean endemic aromatic plants	Global medicinal plants	Aromatic plants in various regions, with focus on spatiotemporal factors
**Taxonomic Level Examined**	Genus- and species-level chemotaxonomy	Species and genus-level chemotaxonomy	Ecological, chemical, and taxonomic scales
**Data Coverage (Number of Species Studied)**	Focus on Korean species	Wide global focus, many species of medicinal plants	Focus on aromatic plants with emphasis on chemical diversity
**Key Findings/Conclusions**	Integrates metabolomics with taxonomy for Korean endemic flora	Highlights the role of alkaloids and terpenoids in plant classification	Focus on chemical phenotypic plasticity and environmental influences on chemical diversity

Table S2: Summary of Bioactivity Studies and Knowledge Gaps in Korean Aromatic Plants

Bioactivity	*In Vivo* Studies	*In Vitro* Studies	Usual Effective Dose Range	Main Knowledge Gaps
**Antimicrobial**	-	45	Variable but generally <64μg/mL)	Limited studies on the synergistic effects of bioactive compounds; lack of dose-response relationships in vivo.
**Anti-inflammatory**	-	55	Variable but generally<64μg/mL)	Insufficient evidence on the long-term safety of extracts; no standardized doses for clinical use.
**Antioxidant**	-	60	Variable but Generally <64μg/mL)	Need for human clinical trials; variability in the chemical composition of extracts affecting outcomes.
**Analgesic**	-	35	Variable but generally <64μg/mL)	Inconsistent findings across studies; lack of detailed molecular mechanisms for pain relief.
**Anticancer**	12	25	Variable	Limited studies on the long-term effectiveness; insufficient data on the bioavailability of active compounds.
